# Review on Antifungal Resistance Mechanisms in the Emerging Pathogen *Candida auris*

**DOI:** 10.3389/fmicb.2019.02788

**Published:** 2019-11-29

**Authors:** Farid Chaabane, Artan Graf, Léonard Jequier, Alix T. Coste

**Affiliations:** ^1^School of Biology, University of Lausanne, Lausanne, Switzerland; ^2^Institute of Microbiology, University Hospital (CHUV), Lausanne, Switzerland

**Keywords:** *Candida auris*, antifungal resistance, biofilm, emerging pathogen, MIC, outbreaks

## Abstract

*Candida auris* is an emerging multi-drug resistant yeast, that causes major issues regarding patient treatment and surface disinfection in hospitals. Indeed, an important proportion of *C. auris* strains isolated worldwide present a decreased sensitivity to multiple and sometimes even all available antifungals. Based on recent tentative breakpoints by the CDC, it appears that in the USA about 90, 30, and < 5% of isolates have been resistant to fluconazole, amphotericin B, and echinocandins, respectively. To date, this has lead to a low therapeutic success. Furthermore, *C. auris* is prone to cause outbreaks, especially since it can persist for weeks in a nosocomial environment and survive high-end disinfection procedures. In this review, we describe the molecular resistance mechanisms to antifungal drugs identified so far in *C. auris* and compare them to those previously discovered in other *Candida* species. Additionally, we examine the role that biofilm formation plays in the reduced antifungal sensitivity of this organism. Finally, we summarize the few insights on how this yeast survives on hospital surfaces and discuss the challenge it presents regarding nosocomial environment disinfection.

## 1. Introduction

**Figure 1:**
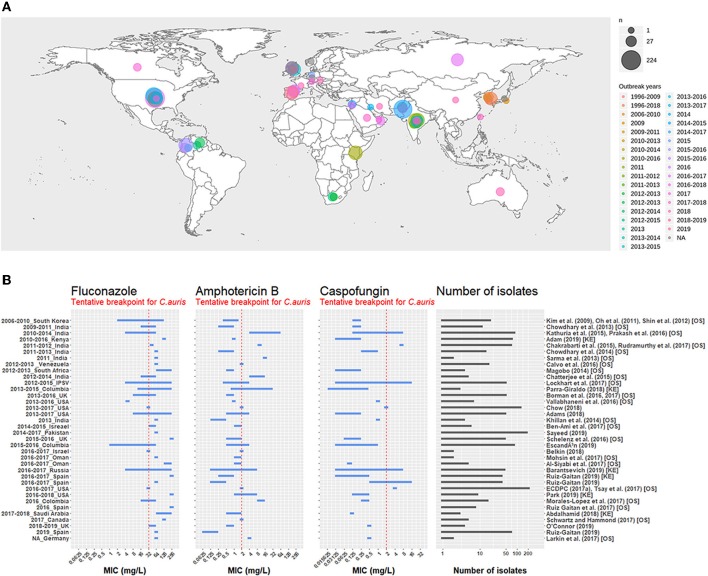
**(A)** Map representing the number of isolates per outbreak since 1996. The first outbreak year is brown-colored and the latest outbreak year is in pink. Circle size is proportional to the number of isolates reported for each outbreak. Outbreak survey was performed recovering data from three main reviews (Osei Sekyere, 2018; Jackson et al., 2019; Kenters et al., 2019) and complemented by a pubmed search using the query “Candida” AND “auris” AND “outbreak” as keywords. Results were limited in time, between the 21/07/2017, the latest study in the systematic review by Osei Sekyere and the 31/08/2019, when the literature search was performed. Studies not present in the review by Osei Sekyere: (Biswal et al., 2017; Abdalhamid et al., 2018; Adams et al., 2018; Belkin et al., 2018; Chow et al., 2018; Desoubeaux et al., 2018; Lesho et al., 2018; Mayor, 2018; Parra-Giraldo et al., 2018; Ruiz-Gaitán et al., 2018, 2019a,b; Sexton et al., 2018; Adam et al., 2019; Barantsevich et al., 2019; Escandón et al., 2019; Leach et al., 2019; O'Connor et al., 2019; Park et al., 2019; Sana et al., 2019; Sayeed et al., 2019). **(B)** MIC ranges for three antifungals measured in *C. auris* isolates from different outbreaks around the world. The red dotted line represents the tentative breakpoint proposed by the CDC. IPSV is an acronym for “India, Pakistan, South Africa, and Venezuela,” as strains from these countries were all analyzed in the same study. Outbreak survey was performed identically to **(A)**. Only outbreak with >1 isolate were plotted. Studies cited by Osei Sekyere are signaled with [OS] and studies cited by Kenters et al. with [KE].

In the last decade, an emerging nosocomial *Candida* species has been causing severe cases of candidemia and other types of fungal infections. Outbreaks of the responsible pathogen, namely *Candida auris*, have been observed all around the world (see Figure 1A) (Lockhart et al., 2017).

Whole genome sequencing determined the presence of four specific clades within the *C. auris* species: East Asia, South Asia, South Africa, and South America. All clades are characterized by distinct single nucleotide polymorphisms (SNPs), which highlights the independent and worldwide emergence of this pathogen (Lockhart et al., 2017).

Comparative studies in different animal models (murine and *Galleria mellonella* larvae) showed strain-dependant virulence levels for *C. auris*. In most cases, *C.auris* strains proved to be less virulent than *C. albicans* (Borman et al., 2016; Ben-Ami et al., 2017; Fakhim et al., 2018; Wang et al., 2018). However, reported cases of *C. auris* outbreaks were linked with a high mortality rate, primarily in immunocompromised patients. Other conditions such as diabetes, cardiovascular and/or pulmonary diseases, sepsis or a prior treatment with antibiotics seemed also to be important risk factors (Osei Sekyere, 2018).

Exceptionally for a yeast, *C. auris* has successfully spread in nosocomial environments. For example, in a UK hospital, a single patient colonized with *C. auris* led to subsequent identifications of other cases among other hospitalized patients. The pathogen was also detected on the healthcare staff even several months after its first isolation, suggesting an efficient human to human transmission. Additionally, traces of *C. auris* had been found on bed sides, windowsills, monitors, and other equipment throughout the hospital, showing that *C. auris* is capable to persist on nosocomial surfaces (Schelenz et al., 2016), in contrast to other *Candida* species. Investigation into an outbreak in another UK hospital reported that the use of reusable equipment such as axillary temperature probes was the primary cause of transmission to patients (Eyre et al., 2018), further illustrating its capability to survive on surfaces.

The capacity to survive in nosocomial environments could be due to the ability of *C. auris* to form biofilms; a growth form in which cells are arranged together in microcolonies protected by a matrix of glucan. By convention, cells enclosed in the matrix are called sessile, and cells outside of it are called planktonic. *C. auris* cells protected by this structure were proven to be difficult to eliminate, even with high-levels of disinfection procedures (Kean et al., 2018b).

In addition to potentially allowing an increased survival on inert surfaces, the ability of *C. auris* to form biofilms may also be an important factor of its resistance to systemic antifungals used in patient treatment. Indeed, the fact that biofilm forming cells were isolated from wounds and catheter tips indicates that some *C. auris* cells could survive in biofilm-form in patients (Borman et al., 2016) and it was shown that sessile cells presented reduced susceptibility for several antifungals. (Sherry et al., 2017). Finally, biofilm has also been shown to be an important virulence factor, as biofilm forming *C. auris* strains are linked to increased morbidity and mortality (Borman et al., 2016).

With this review we aim to highlight molecular resistance mechanisms acquired by *C. auris*, which led to its extraordinary survival in nosocomial environment and in hospitalized patients.

## 2. *C. auris* Antifungal Susceptibility

After 10 years of recording minimum inhibitory concentration (MIC) for available antifungals, the CDC recently published a tentative of breakpoints to better define *C. auris* antifungal susceptibility^1^. It clearly appeared that *C. auris* has exceptionally high MICs for the four main classes of antifungal drugs: azoles, echinocandins, polyenes (Figure 1B), and nucleoside analogs. In this chapter, we will examine in detail two factors that proved to play a role in this decreased susceptibility to antifungal treatments: molecular resistance mechanisms and biofilm-associated resistance mechanisms.

### 2.1. Molecular Resistance Mechanisms

We define here molecular resistance as the ways individual cells can decrease their sensitivity to antifungals due to genetic modifications leading to drug target modification, efflux pumps overexpression, or metabolism modifications (see for review: Pemán et al., 2009; Sanglard et al., 2009; Krishnasamy et al., 2018). This section compiles, for each class of antifungal, the main molecular resistance mechanisms found in *C. auris* up to now. Additionally, Table 1 summarizes for each drug, the mode of action, the related resistance mechanisms described in non-*auris Candida* and the resistance mechanisms discovered in *C. auris*, as of yet.

**Table 1:** Pharmacodynamics of the four main antifungal classes and linked resistance mechanisms in *Candida* spp. in general and *C. auris* specifically.

**Antifungal** ** class**	**Target**	**Effect**	**Resistance mechanism(s)** ** in non-*auris Candida* spp**.	**Resistance mechanism(s) in** ***Candida auris***
Azoles	Inhibits Lanosterol 14-alpha -demethylase (LD), the protein catalyzing the conversion of lanosterol to ergosterol	Weaker cell membrane, inhibiting growth and sensitizing to host immune system Accumulation of toxic intermediary compound	MSF efflux pumps overexpression due to mutation in Mrr1 transcription factor (TF). [a] *CDR* genes overexpression, encoding ABC efflux pumps, due to mutation in Tac1 TF. [a] Point mutation in gene encoding LD (*ERG11*) Upregulation of *ERG11* due to mutation in Upc2 TF. [a] *ERG3* mutation:Bypass ergosterol synthesis pathway to prevent toxic compound accumulation	Presence of MFS efflux pumps genes [b]. Higher ABC efflux pumps activity compared to *C.glabrata*. [c] Significant contribution of **CDR*1* to resistance. [d] *ERG11* point mutation : Y132F ; [f] K143R [f] Presence of *ERG3* [e] but no mutation associated with increased resistance reported
Echinocandin	Inhibits beta(1,3)D-glucan synthase	Defective cell wall formation	Mutation in *FKS1* and *FKS2* gene, encoding the target enzyme	Presence of *FKS1* and *FKS2* In *FKS1* three mutations linked to increased MIC: S639Y; [g] S639P; [g] S639F. [h]
Polyene	Binds ergosterol molecules in the cytoplasmic membrane	Affects membrane permeability by forming multimeric pores and causes oxidative damage	Membrane composition alteration to reduce target exposition Increase catalase activity to counter oxidative damage	5 SNPs in different genomic loci associated with reduced sensitivity. [i]
Nucleoside analogs	Transformed in the fungal cell in two products that respectively inhibit thymidine synthase and get incorporated in RNA	DNA and RNA synthesis inhibition	Modification of enzymes involved in 5-FC uptake or metabolism Competitive inhibition of 5-FC effect by increased pyrimidine synthesis	Amino-acid substitution (F211I) in *FUR1*, a gene involved in 5-FC metabolism found in a single flucytosine- resistant isolate [g]

*Adapted from Krishnasamy et al. (2018). With additional information from: [a] Vandeputte et al. (2012), [b] Sharma et al. (2016), [c] Ben-Ami et al. (2017), [d] Chowdhary et al. (2018), [e] Rybak et al. (2019), [f] Healey et al. (2018), [g] Rhodes et al. (2018), [h] Kordalewska et al. (2018), [i] Escandón et al. (2019)*.

#### 2.1.1. Mechanisms of Resistance to Azoles

##### 2.1.1.1. MFS and ABC efflux pumps overexpression

Efflux pumps are proteins that transport components across the cell membrane. Some of them can pump drugs outside of the cell, lowering their concentration and effect on the cell. There are two major families of efflux pumps involved in antifungal resistance: ATP Binding Cassette (ABC) and Major Facilitator Superfamily (MFS) transporters. Enhanced overexpression of efflux pumps is one of the major resistance mechanism to azoles in pathogenic *Candida* species (Schuetzer-Muehlbauer et al., 2003; Morschhäuser et al., 2007).

Indian strains of *C. auris* sequenced in two different studies exhibit numerous genes orthologous to ABC and MFS transporters of *C. albicans* (Chatterjee et al., 2015; Sharma et al., 2016). Interestingly, *C. auris* strains that were isolated in Israel showed an intrinsicly high ABC transporter activity compared to *C. glabrata* and *C. haemulonii*, by measuring efflux of a fluorescent substrate (Rhodamine 6G) (Ben-Ami et al., 2017).

In *C. albicans, CDR1* is a gene encoding for an ABC efflux pump, known for its role in azole resistance (Sanglard et al., 1995). A gene homologous to *CDR1* was found in *C. auris*. (Rybak et al., 2019). The same study also showed that the deletion of this gene could increase susceptibility of resistant strains from 64- to 128-fold (Rybak et al., 2019).

##### 2.1.1.2. Point mutations in *ERG11*

Ergosterol is a key membrane component in fungi. In *Candida* spp., its biosynthesis is mediated, among others, by the enzyme Lanosterol 14-alpha-demethylase (LD), encoded by the gene *ERG11* which converts lanosterol to ergosterol. The LD is the primary target of azoles, antifungals that inhibit the function of the enzyme and effectively shut down ergosterol biosynthesis, thus impairing membrane integrity (Sanglard et al., 1998).

Point mutations in *ERG11* have been shown to reduce azole sensitivity in *Candida* spp., particularly in three “hot-spot” regions located between amino-acids 105–165, 266–287, and 405–488 (Vandeputte et al., 2012).

Interestingly, after having retrieved the amino acid sequences of 44 *C. auris* isolates from India, 15 missense mutations were found when aligned to the *C. albicans* wild-type *ERG11* sequence (Chowdhary et al., 2018). Five of these mutations were already associated with azole resistance in *C. albicans*. Among these variants two were found in every resistant strain tested in this study, namely Y132F or K143R (Chowdhary et al., 2018). It can also be noted that these mutations are in the first “hot spot” discussed earlier.

A screen for variants in the *ERG11* gene of *C. auris* strains from Columbia identified the same Y132F and K143R substitutions. Upon heterologous expression of these two mutations in *S. cerivisiae*, MICs for azoles presented a two-fold increase as compared to *S. cerevisiae* strains expressing the wild-type *C. auris ERG11* gene (Healey et al., 2018).

##### 2.1.1.3. *ERG11* overexpression

In *C. albicans*, an overexpression of *ERG11* has been linked to a resistance to azole treatment as well.The increased production of LD overwhelms the capacity of the antifungal to inhibit the activity of the protein, resulting in an active protein despite drug treatment (Lopez-Ribot et al., 1998).

In *C. auris*, real time PCR experiments showed that in absence of fluconazole, there was no difference in *ERG11* expression between fluconazole-susceptible and fluconazole-resistant strains (Chowdhary et al., 2018). In presence of the antifungal, *ERG11* expression rises in resistant strains when compared to a control without fluconazole (Chowdhary et al., 2018). However, the effect of this drug on *ERG11* expression was not tested on susceptible strains. Therefore, conditionally increased expression of *ERG11* in presence of fluconazole has not been demonstrated to be specific to resistant strains, until now.

#### 2.1.2. Mechanisms of Resistance to Echinocandins

Beta(1,3)D-glucan is a key component of the fungal cell wall and is encoded by the *FKS1* and *FKS2* genes, two subunits of the Beta(1,3)D-glucan synthase. Echinocandins inhibit this enzyme, decreasing the amount of glucans in the cell wall (Martins et al., 2011).

In *C. albicans* and other non-*auris Candida* species, several mutations leading to echinocandin resistance were found in the same two regions of *FKS1* and *FKS2*. So, they were named “hot-spots” 1 and 2 (HS1 and HS2). In the *C. albicans FKS1* gene these “hot-spots” are located between amino acids 641–649 and 1,345–1,365 (Park et al., 2005). Sequencing of the corresponding hot-spot regions of 38 *C. auris* strains lead to the discovery of an S639F amino acid substitution that is correlated with pan-echinocandin resistance: it was present in all four resistant strains and absent in the 34 susceptible ones. This position in *C. auris FKS1* is aligned to the position 645 (also a Serine) in *C. albicans* (Chowdhary et al., 2018). Interestingly, this mutation is in the region aligning to the HS1 of *C. albicans FKS1*. Other studies observed different mutations at the same location in echinocandin-resistant *C. auris* strains: S639Y and S639P (Rhodes et al., 2018). The latter was confirmed to cause echinocandin resistance *in vivo* in a mouse model (Kordalewska et al., 2018).

*FKS2* was also found in a single copy in the *C. auris* genome (Sharma et al., 2016), but no mutation associated with echinocandin resistance were found in this gene to our knowledge.

#### 2.1.3. Mechanisms of Resistance to Polyenes

As shown in the Figure 1B, several *C. auris* strains are resistant to Amphotericin B (AMB). In the *Candida* species, modifications of the sterol composition of the membrane have been highlighted as a resistance mechanism (Haynes et al., 1996; Nolte et al., 1997). In *C. albicans*, mutations in *ERG 2, 3, 5, 6* or *11* have been shown to have this effect (Arendrup and Patterson, 2017). Rhodes et al. screened 27 *C. auris* isolates from the UK for SNPs in these genes in strains displaying reduced sensitivity to AMB. However, no variants explaining these differences in drug susceptibility were found (Rhodes et al., 2018).

#### 2.1.4. Mechanisms of Resistance to Flucytosine (5-Fluorocytosine)

Flucytosine is a nucleoside analog that inhibits nucleic acid synthesis. After cell entry, flucytosine has to be activated to have an antifungal effect. This activation requires, among others, the protein encoded by the gene *FUR1* Waldorf and Polak (1983). In non-*auris Candida species* mutations in *FUR1* were demonstrated to be linked with flucytosine resistance (Vandeputte et al., 2012).

Rhodes et al. sequenced a flucytosine resistant *C. auris* strain and observed a F211I amino acid substitution in the *FUR1* gene (Rhodes et al., 2018). This specific missense mutation has no known equivalent in other *Candida* species, therefore additional studies are required to determine if this mutation is the cause of the resistance to flucytosine in the tested *C. auris* strain (Rhodes et al., 2018). However, this drug is less employed than other antifungals. Because of that, fewer studies have been performed to better understand the resistance of *C. auris* to this compound.

To summarize, some resistance mechanisms previously discovered in *Candida* species like *C. glabrata* and *C. albicans*, were also showcased by *C. auris*. They are listed in the last column of the Table 1. However, *C. auris* antifungal resistance is only partially explained by these previously described resistance mechanisms. In order to eventually design new therapeutic agents and more generally to improve patient care, further studies are needed to better understand the underlying mechanisms leading to the exceptionally high MIC ranges presented by some *C. auris* strains (see Figure 1B).

### 2.2. Biofilm: An Antifungal Resistant Way of Life

It was shown that MICs for several antifungals are higher in *C. auris* sessile cells (up to 4-, 20-, 60-fold for voriconazole, Amphotericin B, and Micafungin, respectively) compared to planktonic cells (Sherry et al., 2017). Similarly, minimal biofilm eradication concentrations were shown to be 512-fold higher than MICs for echinocandins and azoles (Romera et al., 2019). This phenomenon was previously shown for *C. albicans* (Hawser and Douglas, 1995). The molecular mechanisms responsible for these elevated MIC are still largely unknown in *C. auris* but some studies have already provided some insights.

MSF and ABC efflux pump-encoding genes were found to be upregulated (2- to 4-fold) in sessile cells compared to planktonic cells (Kean et al., 2018a). Concordantly, the activity of corresponding proteins was 2-fold increased. However, when treated with efflux pump inhibitors the antifungal sensitivity of sessile cells was increased 4- to 16-fold after 12 h. This suggests that efflux pumps play an important role in the resistance to antifungals displayed by this type of cells (Kean et al., 2018a).

In *C. albicans*, the exopolymer matrix is known to non-specifically bind to all classes of antifungals and sequestrate them outside the cells (Nett et al., 2010). As *Candida* spp. share a core polysaccharide profile, it was suggested that this mechanism might be conserved in *C. auris* as well (Kean et al., 2018a). This was later confirmed in *in vivo* and *in vitro* assays (Dominguez et al., 2019).

## 3. Nosocomial Persistence

The capacity of *C. auris* to colonize and persist on surfaces is exceptional: it is able to persist longer on moist surfaces in comparison to *C. albicans* (Piedrahita et al., 2017). It also has a prolonged metabolic activity on surfaces, akin to *C. parapsilosis*, a known skin and plastic colonizer (Piedrahita et al., 2017; Welsh et al., 2017).

The increased survival of *C. auris* on surfaces was investigated by two studies. The first claimed it could be linked to an enhanced environmental stress resistance to temperature and other stressors (Kean et al., 2018b). The second hypothesizes that it could be due to the formation of biofilm. However, this remains to be proven as, so far, no biofilm forming isolate was recovered from environmental surfaces (Ku et al., 2018). It is to be noted that these two possibilities are not mutually exclusive, and that biofilm formation might even be the cause of the increased stress resistance (Ku et al., 2018).

The persistence of *C. auris* in nosocomial environments is heightened by the resistance of this pathogen to disinfection procedures. The efficacy of NaOCl and peracetic acid on stainless steel, polymer (polyester coverslips) and cellulose surfaces were tested. Both disinfectants showed a significant efficacy to kill *C. auris* cells on all surfaces. However, some viable cells remained after applying NaOCl on non-porous surfaces (stainless steel and polyester coverslips). In fact, higher concentrations of this disinfectant coupled to longer exposure times were required to lower regrowth, but even then, they were not able to completely eradicate the pathogen. For example, a 10,000 parts per million (ppm) NaOCl concentration coupled to a 5 min exposure on stainless steel, was enough to observe a significant reduction of colonies. However, substantial regrowth was observed after re-inoculation onto a rich medium (Kean et al., 2018b).

For peracetic acid, which is a high-level disinfectant, this regrowth was surface dependent, as no regrowth was observed for polymer as opposed to stainless steel. They performed the same experiments on *C. glabrata* and *C. albicans* and obtained similar results (Kean et al., 2018b).

This shows that high-level disinfection procedures may be insufficient for a complete surface cleansing and that the pre-established guidelines for surface disinfection have to be adapted to these *Candida* species (Kean et al., 2018b). Factors like the type of surfaces and exposure times should be considered when implementing these disinfection procedures (Kean et al., 2018b; Ku et al., 2018) not only cost and ease of use, as is it often the case (Ku et al., 2018).

Based on the results of two studies (Cadnum et al., 2017; Rutala et al., 2019), the CDC recommends using disinfectants effective against *Clostridioides difficile* spores. If not possible, they suggest alternatives such as hydrogen peroxyde 0.5–1.4% or quaternary ammonium compounds supplemented with isopropyl alcohol and/or ethyl alcohol^2^. Finally, ultraviolet-C light could also be an interesting candidate for surfaces disinfection. It proved highly effective at killing *C. auris* colonies, given sufficient exposure time and distance. However, further studies would be necessary before implementing such methods in hospital settings (de Groot et al., 2019).

## 4. Conclusion

The pathogenic and virulent capacities of *C. auris* are profoundly worrying. Especially since outbreaks occurred independently in different regions of the world and that the associated isolates showed decreased susceptibility to the most commonly used antifungal drugs used for patient treatment (see Figure 1).

To explain the reduced antifungal susceptibility in *C. auris*, researchers made use of the extended knowledge already available on other *Candida* species, particularly *C. albicans*. As a result, some of the resistance mechanisms of *C. auris* were highlighted (see Table 1). More precisely, two point mutations in *ERG11* and the overexpression of the ABC transporter Cdr1 were proven to decrease fluconazole sensitivity. Furthermore, an amino acid substitution in *FKS1* was shown to reduce the sensitivity of *C. auris* to echinocandins, which is normally the drug of choice for treatment against this pathogen (Bidaud et al., 2018). Finally, a mutation in *FUR1* was identified in a flucytosine resistant *C. auris* strain, but remains to be demonstrated as the cause of this reduced sensitivity. Biofilm is thought to be a resistance mechanism *per se* as it was shown to decrease drug susceptibility. However, further research is still necessary to describe the whole variety of processes that take place in this growth form. It is important to note that biofilm is a complex structure that remains poorly understood and difficult to investigate, even in well-known species such as *C. albicans*.

The resistance properties of *C. auris* are coupled to a propensity to cause nosocomial infections. Its ability to adhere to a variety of nosocomial surfaces and survive disinfection are critical propagation factors. The presence of multiple types of surfaces poses a challenge for clinical institutions, as standard disinfection procedures are inefficient and unadapted for each type of them. This problem, also arises from the insufficient research on this subject. In addition, a better understanding of the mechanisms underlying *C. auris* disinfectant resistance would help to improve hygiene protocols and avoid future nosocomial outbreaks.

## Author Contributions

FC, AG, and LJ participated equally in the redaction of this review. AC supervised and participated in the redaction of this review.

### Conflict of Interest

The authors declare that the research was conducted in the absence of any commercial or financial relationships that could be construed as a potential conflict of interest.

## Footnotes

^1^CDC (2019). Antifungal Susceptibility Testing and Interpretation. Available online at: https://www.cdc.gov/fungal/candida-auris/c-auris-antifungal.html (accessed April 29, 2019).

^2^CDC (2018). Infection Prevention and Control for *Candida auris*. Available online at: https://www.cdc.gov/fungal/candida-auris/c-auris-infection-control.html (accessed November 12, 2019).

## References

[B1] Abdalhamid B. Almaghrabi R. Althawadi S. Omrani A. (2018). First report of *Candida auris* infections from Saudi Arabia. J. Infect. Public Health 11, 598–599. 10.1016/j.jiph.2018.05.010 29895475

[B2] Adam R. D. Revathi G. Okinda N. Fontaine M. Shah J. Kagotho E.. (2019). Analysis of *Candida auris* fungemia at a single facility in Kenya. Int. J. Infect. Dis. 85, 182–187. 10.1016/j.ijid.2019.06.001 31185293

[B3] Adams E. Quinn M. Tsay S. Poirot E. Chaturvedi S. Southwick K. (2018). *Candida auris* in healthcare facilities, New York, USA, 2013-2017. Emerg. Infect. Dis. 24, 1816–1824. 10.3201/eid2410.180649 30226155 PMC6154128

[B4] Arendrup M. C. Patterson T. F. (2017). Multidrug-resistant candida: epidemiology, molecular mechanisms, and treatment. J. Infect. Dis. 216, S445–S451. 10.1093/infdis/jix131 28911043

[B5] Barantsevich N. E. Orlova O. E. Shlyakhto E. V. Johnson E. M. Woodford N. Lass-Floerl C.. (2019). Emergence of *Candida auris* in Russia. J. Hosp. Infect. 102, 445–448. 10.1016/j.jhin.2019.02.021 30851375

[B6] Belkin A. Gazit Z. Keller N. Ben-Ami R. Wieder-Finesod A. Novikov A.. (2018). *Candida auris* infection leading to nosocomial transmission, Israel, 2017. Emerg. Infect. Dis. 24, 801–804. 10.3201/eid2404.171715 29553329 PMC5875262

[B7] Ben-Ami R. Berman J. Novikov A. Bash E. Shachor-Meyouhas Y. Zakin S.. (2017). Multidrug-resistant *Candida haemulonii* and *C. auris*, Tel Aviv, Israel. Emerg. Infect. Dis. 23, 195–203. 10.3201/eid2302.161486 28098529 PMC5324804

[B8] Bidaud A. L. Chowdhary A. Dannaoui E. (2018). *Candida auris*: an emerging drug resistant yeast – A mini-review. J. Mycol. Méd. 28, 568–573. 10.1016/j.mycmed.2018.06.007 30030072

[B9] Biswal M. Rudramurthy S. M. Jain N. Shamanth A. S. Sharma D. Jain K.. (2017). Controlling a possible outbreak of *Candida auris* infection: lessons learnt from multiple interventions. J. Hosp. Infect. 97, 363–370. 10.1016/j.jhin.2017.09.009 28939316

[B10] Borman A. M. Szekely A. Johnson E. M. (2016). Comparative pathogenicity of United Kingdom isolates of the emerging pathogen *Candida auris* and other key pathogenic candida species. mSphere 1:e00189–16. 10.1128/mSphere.00189-16 27547827 PMC4990711

[B11] Cadnum J. L. Shaikh A. A. Piedrahita C. T. Sankar T. Jencson A. L. Larkin E. L.. (2017). Effectiveness of disinfectants against *Candida auris* and other candida species. Infect. Control Hosp. Epidemiol. 38, 1240–1243. 10.1017/ice.2017.162 28793937

[B12] Chatterjee S. Alampalli S. V. Nageshan R. K. Chettiar S. T. Joshi S. Tatu U. S. (2015). Draft genome of a commonly misdiagnosed multidrug resistant pathogen *Candida auris*. BMC Genomics 16:686. 10.1186/s12864-015-1863-z 26346253 PMC4562351

[B13] Chow N. A. Gade L. Tsay S. V. Forsberg K. Greenko J. A. Southwick K. L.. (2018). Multiple introductions and subsequent transmission of multidrug-resistant *Candida auris* in the USA: a molecular epidemiological survey. Lancet Infect. Dis. 18, 1377–1384. 10.1016/S1473-3099(18)30597-8 30293877 PMC6556114

[B14] Chowdhary A. Prakash A. Sharma C. Kordalewska M. Kumar A. Sarma S.. (2018). A multicentre study of antifungal susceptibility patterns among 350 *Candida auris* isolates (2009–17) in India: role of the ERG11 and FKS1 genes in azole and echinocandin resistance. J. Antimicrob. Chemother. 73, 891–899. 10.1093/jac/dkx480 29325167

[B15] de Groot T. Chowdhary A. Meis J. F. Voss A. (2019). Killing of *Candida auris* by UV-C: importance of exposure time and distance. Mycoses 62, 408–412. 10.1111/myc.12903 30748018 PMC6850319

[B16] Desoubeaux G. Bailly E. Guillaume C. De Kyvon M.-A. Tellier A.-C. Morange V.. (2018). *Candida auris* in contemporary mycology labs: a few practical tricks to identify it reliably according to one recent French experience. J. Mycol. Med. 28, 407–410. 10.1016/j.mycmed.2018.02.011 29567284

[B17] Dominguez E. G. Zarnowski R. Choy H. L. Zhao M. Sanchez H. Nett J. E.. (2019). Conserved role for biofilm matrix polysaccharides in *Candida auris* drug resistance. mSphere 4:e00680–18. 10.1128/mSphereDirect.00680-18 30602527 PMC6315084

[B18] Escandón P. Chow N. A. Caceres D. H. Gade L. Berkow E. L. Armstrong P. (2019). Molecular epidemiology of *Candida auris* in colombia reveals a highly related, countrywide colonization with regional patterns in amphotericin B resistance. Clin. Infect. Dis. 68, 15–21. 10.1093/cid/ciy411 29788045

[B19] Eyre D. W. Sheppard A. E. Madder H. Moir I. Moroney R. Quan T. P.. (2018). A *Candida auris* outbreak and its control in an intensive care setting. N. Engl. J. Med. 379, 1322–1331. 10.1056/NEJMoa1714373 30281988

[B20] Fakhim H. Vaezi A. Dannaoui E. Chowdhary A. Nasiry D. Faeli L.. (2018). Comparative virulence of Candida auris with Candida haemulonii, Candida glabrata and Candida albicans in a murine model. Mycoses 61, 377–382. 10.1111/myc.12754 29460345

[B21] Hawser S. P. Douglas L. J. (1995). Resistance of *Candida albicans* biofilms to antifungal agents *in vitro*. Antimicrob. Agents Chemother. 39, 2128–2131. 10.1128/AAC.39.9.2128 8540729 PMC162894

[B22] Haynes M. P. Chong P. L.-G. Buckley H. R. Pieringer R. A. (1996). Fluorescence studies on the molecular action of amphotericin B on susceptible and resistant fungal cells. Biochemistry 35, 7983–7992. 10.1021/bi952910c 8672502

[B23] Healey K. R. Kordalewska M. Ortigosa C. J. Singh A. Berrío I. Chowdhary A.. (2018). Limited ERG11 mutations identified in isolates of *Candida auris* directly contribute to reduced azole susceptibility. Antimicrob. Agents Chemother. 62, e01427–18. 10.1128/AAC.01427-18 30082281 PMC6153782

[B24] Jackson B. R. Chow N. Forsberg K. Litvintseva A. P. Lockhart S. R. Welsh R.. (2019). On the origins of a species: what might explain the rise of *Candida auris*? J. Fungi 5:E58. 10.3390/jof5030058 31284576 PMC6787658

[B25] Kean R. Delaney C. Sherry L. Borman A. Johnson E. M. Richardson M. D.. (2018a). Transcriptome assembly and profiling of *Candida auris* reveals novel insights into biofilm-mediated resistance. mSphere 3:e00334–18. 10.1128/mSphere.00334-18 29997121 PMC6041501

[B26] Kean R. Sherry L. Townsend E. McKloud E. Short B. Akinbobola A.. (2018b). Surface disinfection challenges for *Candida auris*: an *in-vitro* study. J. Hosp. Infect. 98, 433–436. 10.1016/j.jhin.2017.11.015 29203448

[B27] Kenters N. Kiernan M. Chowdhary A. Denning D. W. Pemán J. Saris K. (2019). Control of *Candida auris* in healthcare institutions. Outcome of an ISAC expert meeting. Int. J. Antimicrob. Agents 54, 400–406. 10.1016/j.ijantimicag.2019.08.013 31419480

[B28] Kordalewska M. Lee A. Park S. Berrio I. Chowdhary A. Zhao Y.. (2018). Understanding echinocandin resistance in the emerging pathogen *Candida auris*. Antimicrob. Agents Chemother. 62:e00238–18. 10.1128/AAC.00238-18 29632013 PMC5971591

[B29] Krishnasamy L. Krishnakumar S. Kumaramanickavel G. Saikumar C. (2018). Molecular mechanisms of antifungal drug resistance in Candida species. J. Clin. Diagn. Res. 12, DE01–DE06. 10.7860/JCDR/2018/36218.11961

[B30] Ku T. S. N. Walraven C. J. Lee S. A. (2018). *Candida auris*: disinfectants and implications for infection control. Front. Microbiol. 9:726 10.3389/fmicb.2018.00726 29706945 PMC5906573

[B31] Leach L. Russell A. Zhu Y. Chaturvedi S. Chaturvedi V. (2019). A rapid and automated sample-to-result *Candida auris* real-time PCR assay for high-throughput testing of surveillance samples with BD MAX™ open system. J. Clin. Microbiol. 57:e00630–19. 10.1128/JCM.00630-19 31391229 PMC6760950

[B32] Lesho E. P. Bronstein M. Z. McGann P. Stam J. Kwak Y. Maybank R.. (2018). Importation, mitigation, and genomic epidemiology of *Candida auris* at a large teaching hospital. Infect. Control Hosp. Epidemiol. 39, 53–57. 10.1017/ice.2017.231 29208056

[B33] Lockhart S. R. Etienne K. A. Vallabhaneni S. Farooqi J. Chowdhary A. Govender N. P.. (2017). Simultaneous emergence of multidrug-resistant *Candida auris* on 3 continents confirmed by whole-genome sequencing and epidemiological analyses. Clin. Infect. Dis. 64, 134–140. 10.1093/cid/ciw691 27988485 PMC5215215

[B34] Lopez-Ribot J. L. McAtee R. K. Lee L. N. Kirkpatrick W. R. White T. C. Sanglard D.. (1998). Distinct patterns of gene expression associated with development of fluconazole resistance in serial *Candida albicans* isolates from human immunodeficiency virus-infected patients with oropharyngeal candidiasis. Antimicrob. Agents Chemother. 42, 2932–2937. 10.1128/AAC.42.11.2932 9797228 PMC105968

[B35] Martins I. M. Cortés J. C. G. Muñoz J. Moreno M. B. Ramos M. Clemente-Ramos J. A.. (2011). Differential activities of three families of specific beta(1,3)glucan synthase inhibitors in wild-type and resistant strains of fission yeast. J. Biol. Chem. 286, 3484–3496. 10.1074/jbc.M110.174300 21115488 PMC3030354

[B36] Mayor S. (2018). *Candida auris* outbreak was linked to reusable axillary temperature probes, study finds. BMJ 363:k4133. 10.1136/bmj.k4133 30275113

[B37] Morschhäuser J. Barker K. S. Liu T. T. Bla-Warmuth J. Homayouni R. Rogers P. D. (2007). The transcription factor Mrr1p controls expression of the MDR1 efflux pump and mediates multidrug resistance in *Candida albicans*. PLoS Pathog. 3:e164. 10.1371/journal.ppat.0030164 17983269 PMC2048531

[B38] Nett J. E. Crawford K. Marchillo K. Andes D. R. (2010). Role of Fks1p and matrix glucan in *Candida albicans* biofilm resistance to an echinocandin, pyrimidine, and polyene. Antimicrob. Agents Chemother. 54, 3505–3508. 10.1128/AAC.00227-10 20516280 PMC2916329

[B39] Nolte F. S. Parkinson T. Falconer D. J. Dix S. Williams J. Gilmore C.. (1997). Isolation and characterization of fluconazole- and amphotericin B-resistant *Candida albicans* from blood of two patients with leukemia. Antimicrob. Agents Chemother. 41, 196–199. 10.1128/AAC.41.1.196 8980781 PMC163686

[B40] O'Connor C. Bicanic T. Dave J. Evans T. J. Moxey P. Adamu U.. (2019). *Candida auris* outbreak on a vascular ward - the unexpected arrival of an anticipated pathogen. J. Hosp. Infect. 103, 106–108. 10.1016/j.jhin.2019.06.002 31220481

[B41] Osei Sekyere J. (2018). *Candida auris*: a systematic review and meta-analysis of current updates on an emerging multidrug-resistant pathogen. MicrobiologyOpen 7:e00578. 10.1002/mbo3.578 29345117 PMC6079168

[B42] Park J. Y. Bradley N. Brooks S. Burney S. Wassner C. (2019). Management of patients with *Candida auris* fungemia at community hospital, Brooklyn, New York, USA, 2016–20181. Emerg. Infect. Dis. 25, 601–602. 10.3201/eid2503.180927 30789336 PMC6390749

[B43] Park S. Kelly R. Kahn J. N. Robles J. Hsu M.-J. Register E. (2005). Specific substitutions in the echinocandin target Fks1p account for reduced susceptibility of rare laboratory and clinical *Candida* sp. isolates. Antimicrob. Agents Chemother. 49, 3264–3273. 10.1128/AAC.49.8.3264-3273.2005 16048935 PMC1196231

[B44] Parra-Giraldo C. M. Valderrama S. L. Cortes-Fraile G. Garzón J. R. Ariza B. E. Morio F.. (2018). First report of sporadic cases of *Candida auris* in Colombia. Int. J. Infect. Dis. 69, 63–67. 10.1016/j.ijid.2018.01.034 29421668

[B45] Pemán J. Cantón E. Espinel-Ingroff A. (2009). Antifungal drug resistance mechanisms. Expert Rev. Anti Infect. Ther. 7, 453–460. 10.1586/eri.09.18 19400764

[B46] Piedrahita C. T. Cadnum J. L. Jencson A. L. Shaikh A. A. Ghannoum M. A. Donskey C. J. (2017). Environmental surfaces in healthcare facilities are a potential source for transmission of *Candida auris* and other candida species. Infect. Control Hosp. Epidemiol. 38, 1107–1109. 10.1017/ice.2017.127 28693657

[B47] Rhodes J. Abdolrasouli A. Farrer R. A. Cuomo C. A. Aanensen D. M. Armstrong-James D. (2018). Genomic epidemiology of the UK outbreak of the emerging human fungal pathogen *Candida auris*. Emerg. Microbes Infect. 7:43 10.1101/201343 29593275 PMC5874254

[B48] Romera D. Aguilera-Correa J. J. Gadea I. Viñuela-Sandoval L. García-Rodríguez J. Esteban J. (2019). Candida auris: a comparison between planktonic and biofilm susceptibility to antifungal drugs. J. Med. Microbiol. 68, 1353–1358. 10.1099/jmm.0.001036 31271350

[B49] Ruiz-Gaitán A. Martínez H. Moret A. M. Calabuig E. Tasias M. Alastruey-Izquierdo A.. (2019a). Detection and treatment of *Candida auris* in an outbreak situation: risk factors for developing colonization and candidemia by this new species in critically ill patients. Expert Rev. Anti Infect. Ther. 17, 295–305. 10.1080/14787210.2019.1592675 30922129

[B50] Ruiz-Gaitán A. Moret A. M. Tasias-Pitarch M. Aleixandre-López A. I. Martínez-Morel H. Calabuig E.. (2018). An outbreak due to *Candida auris* with prolonged colonisation and candidaemia in a tertiary care European hospital. Mycoses 61, 498–505. 10.1111/myc.12781 29655180

[B51] Ruiz-Gaitán A. C. Cantón E. Fernández-Rivero M. E. Ramírez P. Pemán J. (2019b). Outbreak of *Candida auris* in Spain: a comparison of antifungal activity by three methods with published data. Int. J. Antimicrob. Agents 53, 541–546. 10.1016/j.ijantimicag.2019.02.005 30769198

[B52] Rutala W. A. Kanamori H. Gergen M. F. Sickbert-Bennett E. E. Weber D. J. (2019). Susceptibility of *Candida auris* and *Candida albicans* to 21 germicides used in healthcare facilities. Infect. Control Hosp. Epidemiol. 40, 380–382. 10.1017/ice.2019.1 30767810

[B53] Rybak J. M. Doorley L. A. Nishimoto A. T. Barker K. S. Palmer G. E. Rogers P. D. (2019). Abrogation of triazole resistance upon deletion of CDR1 in a clinical isolate of *Candida auris*. Antimicrob. Agents Chemother. 63, e00057–19. 10.1128/AAC.00057-19 30718246 PMC6437491

[B54] Sana F. Hussain W. Zaman G. Satti L. Khurshid U. Khadim M. T. (2019). *Candida auris* outbreak report from Pakistan: a success story of infection control in ICUs of a tertiary care hospital. J. Hosp. Infect. 103, 108–110. 10.1016/j.jhin.2019.06.011 31233850

[B55] Sanglard D. Coste A. Ferrari S. (2009). Antifungal drug resistance mechanisms in fungal pathogens from the perspective of transcriptional gene regulation. FEMS Yeast Res. 9, 1029–1050. 10.1111/j.1567-1364.2009.00578.x 19799636

[B56] Sanglard D. Ischer F. Koymans L. Bille J. (1998). Amino acid substitutions in the cytochrome P-450 lanosterol 14-demethylase (CYP51a1) from azole-resistant *Candida albicans* clinical isolates contribute to resistance to azole antifungal agents. Antimicrob. Agents Chemother. 42, 241–253. 9527767 10.1128/aac.42.2.241 PMC105395

[B57] Sanglard D. Kuchler K. Ischer F. Pagani J. L. Monod M. Bille J. (1995). Mechanisms of resistance to azole antifungal agents in *Candida albicans* isolates from AIDS patients involve specific multidrug transporters. Antimicrob. Agents Chemother. 39, 2378–2386. 10.1128/AAC.39.11.2378 8585712 PMC162951

[B58] Sayeed M. A. Farooqi J. Jabeen K. Awan S. Mahmood S. F. (2019). Clinical spectrum and factors impacting outcome of *Candida auris*: a single center study from Pakistan. BMC Infect. Dis. 19:384. 10.1186/s12879-019-3999-y 31060514 PMC6501321

[B59] Schelenz S. Hagen F. Rhodes J. L. Abdolrasouli A. Chowdhary A. Hall A.. (2016). First hospital outbreak of the globally emerging *Candida auris* in a European hospital. Antimicrob. Resist. Infect. Control 5:35. 10.1186/s13756-016-0132-5 27777756 PMC5069812

[B60] Schuetzer-Muehlbauer M. Willinger B. Egner R. Ecker G. Kuchler K. (2003). Reversal of antifungal resistance mediated by ABC efflux pumps from *Candida albicans* functionally expressed in yeast. Int. J. Antimicrob. Agents 22, 291–300. 10.1016/S0924-8579(03)00213-9 13678837

[B61] Sexton D. J. Kordalewska M. Bentz M. L. Welsh R. M. Perlin D. S. Litvintseva A. P. (2018). Direct detection of emergent fungal pathogen *Candida auris* in clinical skin swabs by SYBR green-based quantitative PCR assay. J. Clin. Microbiol. 56:e01337–18. 10.1128/JCM.01337-18 30232130 PMC6258843

[B62] Sharma C. Kumar N. Pandey R. Meis J. F. Chowdhary A. (2016). Whole genome sequencing of emerging multidrug resistant *Candida auris* isolates in India demonstrates low genetic variation. New Microbes New Infect. 13, 77–82. 10.1016/j.nmni.2016.07.003 27617098 PMC5006800

[B63] Sherry L. Ramage G. Kean R. Borman A. Johnson E. M. Richardson M. D.. (2017). Biofilm-forming capability of highly virulent, multidrug-resistant *Candida auris*. Emerg. Infect. Dis. 23, 328–331. 10.3201/eid2302.161320 28098553 PMC5324806

[B64] Vandeputte P. Ferrari S. Coste A. T. (2012). Antifungal resistance and new strategies to control fungal infections. Int. J. Microbiol. 2012:713687. 10.1155/2012/713687 22187560 PMC3236459

[B65] Waldorf A. R. Polak A. (1983). Mechanisms of action of 5-fluorocytosine. Antimicrob. Agents Chemother. 23, 79–85. 10.1128/AAC.23.1.79 6338821 PMC184621

[B66] Wang X. Bing J. Zheng Q. Zhang F. Liu J. Yue H.. (2018). The first isolate of Candida auris in China: clinical and biological aspects. Emerging Microbes & Infections 7, 1–9. 10.1038/s41426-018-0095-0 29777096 PMC5959928

[B67] Welsh R. M. Bentz M. L. Shams A. Houston H. Lyons A. Rose L. J.. (2017). Survival, persistence, and isolation of the emerging multidrug-resistant pathogenic yeast *Candida auris* on a plastic health care surface. J. Clin. Microbiol. 55, 2996–3005. 10.1128/JCM.00921-17 28747370 PMC5625385

